# The risk of falls among the aging population: A systematic review and meta-analysis

**DOI:** 10.3389/fpubh.2022.902599

**Published:** 2022-10-17

**Authors:** Qingmei Xu, Xuemei Ou, Jinfeng Li

**Affiliations:** Department of Geriatrics, The Affiliated Traditional Chinese Medicine Hospital of Southwest Medical University, Luzhou, China

**Keywords:** age, malnutrition, fall, meta-analysis, rural

## Abstract

**Aim:**

This study aims to clarify the risk factors for falls to prevent severe consequences in older adults.

**Methods:**

We searched the PubMed, Web of Science, Embase, and Google Scholar databases using the terms “risk factors” OR “predicting factors” OR “predictor” AND “fall” OR “drop” to identify all relevant studies and compare their results. The study participants were divided into two groups, the “fall group” and the “control group”, and differences in demographic characteristics, lifestyles, and comorbidities were compared.

**Results:**

We included 34 articles in the analysis and analyzed 22 factors. Older age, lower education level, polypharmacy, malnutrition, living alone, living in an urban area, smoking, and alcohol consumption increased the risk of falls in the aging population. Additionally, comorbidities such as cardiac disease, hypertension, diabetes, stroke, frailty, previous history of falls, depression, Parkinson's disease, and pain increased the risk of falls.

**Conclusion:**

Demographic characteristics, comorbidities, and lifestyle factors can influence the risk of falls and should be taken into consideration.

## Introduction

By 2050, people older than 65 years are estimated to account for 16% of the population (1). Falls are a major public health problem, as approximately 28–35% of individuals aged ≥ 65 years experience falls each year. As the aging population increases, more individuals will be at risk of falling (2).Among older people, physical falls are events that adversely affect health and lead to disability and mortality (3, 4). Moreover, fall-associated economic burdens are substantial and continue to increase worldwide (4, 5). Even non-injury falls are associated with negative impacts, such as anxiety, depression, and decreased mobility, which greatly affect the quality of life (QOL) and aging trajectory. The most harmful consequences of injurious falls are hip fracture and brain damage (4). Research on the risk of falling has become increasingly important to maintain the health of older individuals (2).Early screening for the risk of fall that takes risk factors into account is needed. Many retrospective, cross-sectional, and longitudinal studies have examined fall prevalence, fall-related consequences, and risk factors for falls in older individuals. However, even though some reviews have addressed these topics (6, 7), a high-quality systematic review has yet to be conducted. Therefore, in this study, we aimed to investigate the association between lifestyle factors and fall risk in aging adults to promote the development of effective fall prevention strategies.

## Methods

### Guidelines and ethical review

We followed the Preferred Reporting Items for Systematic Reviews and Meta-Analyses (PRISMA) guidelines in this systematic review. As this study was a review, no ethical approval was necessary.

### Search strategy and data extraction

We hypothesized that demographic characteristics, lifestyle factors, and comorbidities would influence the risk of falls in the aging population. We chose these risk factors on the basis of records in the literature. After searching and carefully reading the literature, we found that the above factors had the most related studies and received the most attention. Therefore, we compared these factors between fall and non-fall groups. We searched for potentially relevant articles published in English before January 2022 during the initial search process. The terms searched in the PubMed, Web of Science, Embase, and Google Scholar databases were as follows: “risk factors” OR “predicting factors” OR “predictor” AND “fall” OR “drop”. Since Boolean operators do not work on Google Scholar, we used search terms like “risk factors for fall” and “predicting factors for fall” on Google Scholar. Two authors independently screened all the abstracts and citations of all studies identified with the search strategy to determine eligible studies. Data were independently extracted by two of the authors using a standardized Excel file. Studies were considered eligible if they included two groups and aging individuals (≥65 years old) with or without falls, and presented data on the baseline lifestyle characteristics and comorbidities of the participants. The exclusion criteria were as follows: duplicate publications, reviews, studies on unrelated topics, studies with different variables, and studies with different group criteria. The search process consisted of 2 steps, the initial search with short keywords and then detailed search with detailed search strategy (present in Supplementary File 1). The description of the detailed search strategy for each part of the PICO research question is provided in Supplementary File 1, which is amended for other databases using database-specific subject headings, where available, and keywords in both titles and abstracts. The extracted data included baseline characteristics, lifestyle habits, comorbidities, and occurrence of falls. All the included data were subsequently entered in RevMan 5.1.4.

### Comparisons

In our meta-analysis, we compared 22 factors between the two groups (the fall group and the control [no falls] group). The factors included age, body mass index (BMI), education level, polypharmacy, sex, relationship status (living alone), residential location (rural), (mal)nutrition, smoking status, alcohol consumption, and comorbidities including cardiac disease, hypertension, diabetes, stroke, depression, Parkinson's disease, pain, vision impairment, frailty, previous history of falls, and cognitive impairment.

### Quality assessment

The quality of the included studies was assessed by two authors according to the Cochrane Collaboration Reviewer's Handbook and the Quality of Reporting of Meta-analysis guidelines (40, 41).

### Data analysis

The data were analyzed using RevMan 5.1.4. Continuous outcomes are presented as weighted mean differences (MDs) with 95% confidence intervals (CIs). Dichotomous data are presented as relative risks (RRs) with 95% CIs. A meta-analysis was performed using fixed-effect or random-effects models as appropriate. Specifically, the fixed-effects models were used when no significant heterogeneity was present, and the random-effects models were used when heterogeneity was present. Statistical heterogeneity among the trials was evaluated by the *I*^2^ test, with significance set at *P* < 0.05.

## Results

### Description of the included studies

**Figure 1:**
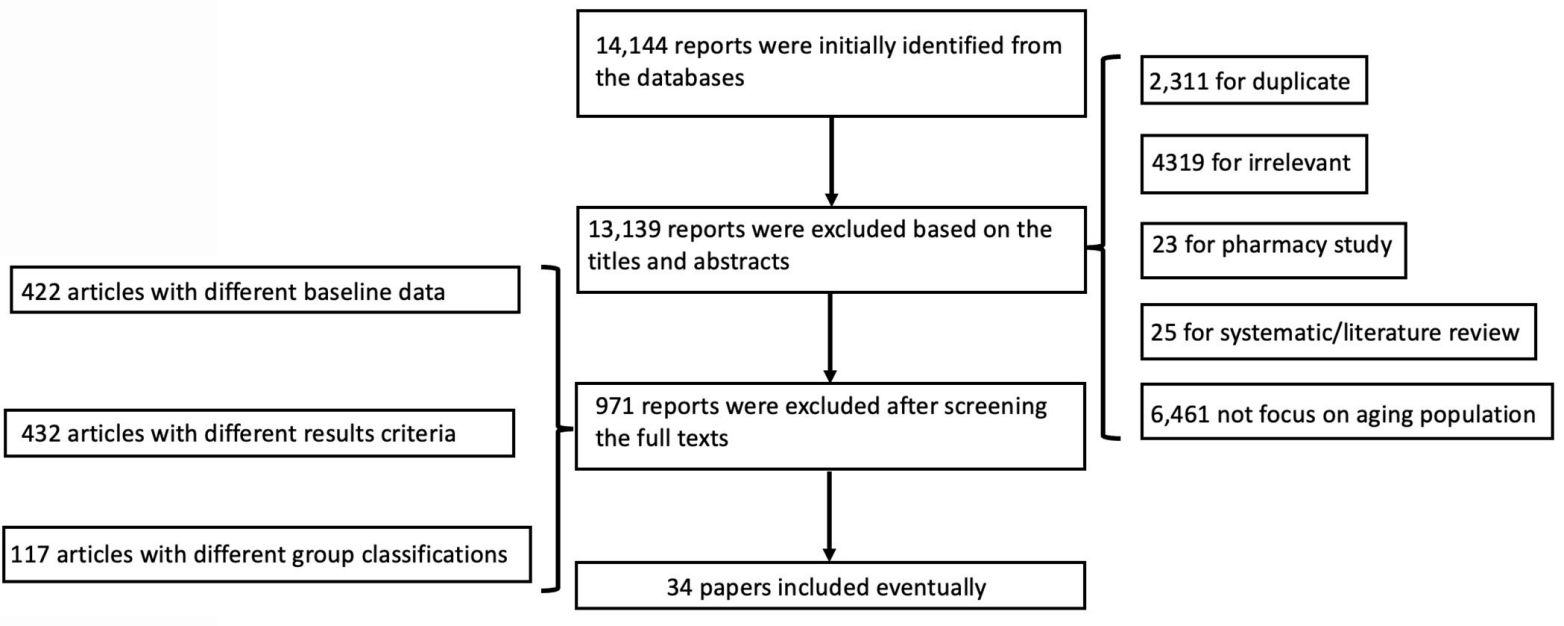
Flowchart of the literature review process and exclusion criteria.

A total of 14,144 reports were initially identified from the databases. After screening for duplicate publications, reviews, and irrelevant records based on the titles and abstracts, 13,139 reports were excluded from the study. After screening the full texts, 422 articles with different baseline data, 432 articles with different results criteria, and 117 articles with different group classifications were excluded. Thus, we eventually included 34 articles in the final analysis (8–32, 34–39, 42–44). The conditions of these studies and the clinical details of the participants are presented in Table 1. A flow chart of the literature search is shown in Figure 1.

**Table 1:** Details of included papers.

**Author**	**Year**	**Included** **number**	**Research type**
**Carvalho**	2020	131	Retrospect study
Díaz et al. (8)	2020	2,849	Retrospect study
Dixe et al. (9)	2021	204	Prospective cohort study
Djurovic et al. (10)	2021	561	Retrospect study
Fukui et al. (11)	2021	185	Prospective cohort study
Griffin et al. (12)	2020	353	Observational study of RCT
Lackoff et al. (13)	2020	2,114	Prospective cohort study
Ilhan et al. (14)	2019	1,441	Retrospect study
Naharci et al. (15)	2020	520	Prospective cohort study
Immonen et al. (16)	2020	872	Retrospect study
Inacio et al. (17)	2021	32,316	Retrospect study
Ishida et al. (18)	2020	6,081	Retrospect study
Kim et al. (19)	2013	294	Retrospect study
Kitayuguchi et al. (20)	2021	965	Prospective cohort study
Pradeep Kumar et al. (21)	2021	63	Cross-sectional study
Pradeep Kumar et al. (21)	2021	150	Retrospect study
Ie et al. (22)	2021	343	Retrospect study
Lee et al. (23)	2021	232	Prospective cohort study
Magnuszewski et al. (24)	2020	358	Cross-sectional study
Makino et al. (25)	2021	2,520	Prospective cohort study
Mat et al. (26)	2021	605	Prospective cohort study
Nugraha et al. (27)	2021	154	Prospective cohort study
Pelicioni et al. (28)	2021	95	Randomized controlled trial
Pereira et al. (29)	2021	508	Cross-sectional study
Ravindran et al. (30)	2016	501	Prospective cohort study
Rivan et al. (31)	2021	815	Prospective cohort study
Sagawa et al. (32)	2018	1,817	Prospective cohort study
Schultz et al. (33)	2015	278	Retrospect study
Severo et al. (34)	2018	358	Prospective cohort study
Teoh et al. (35)	2020	1,415	Cross-sectional study
Tsai et al. (36)	2021	6,153	Retrospect study
Wang et al. (37)	2020	2,049	Prospective cohort study
Yu et al. (38)	2021	237	Prospective cohort study
Yu et al. (38)	2021	1,164	Retrospect study
Zhang et al. (39)	2021	7,307	Retrospect study

RCT, Randomized controlled trial.

### Characteristics and lifestyles of people with/without falls

**Figure 2:**
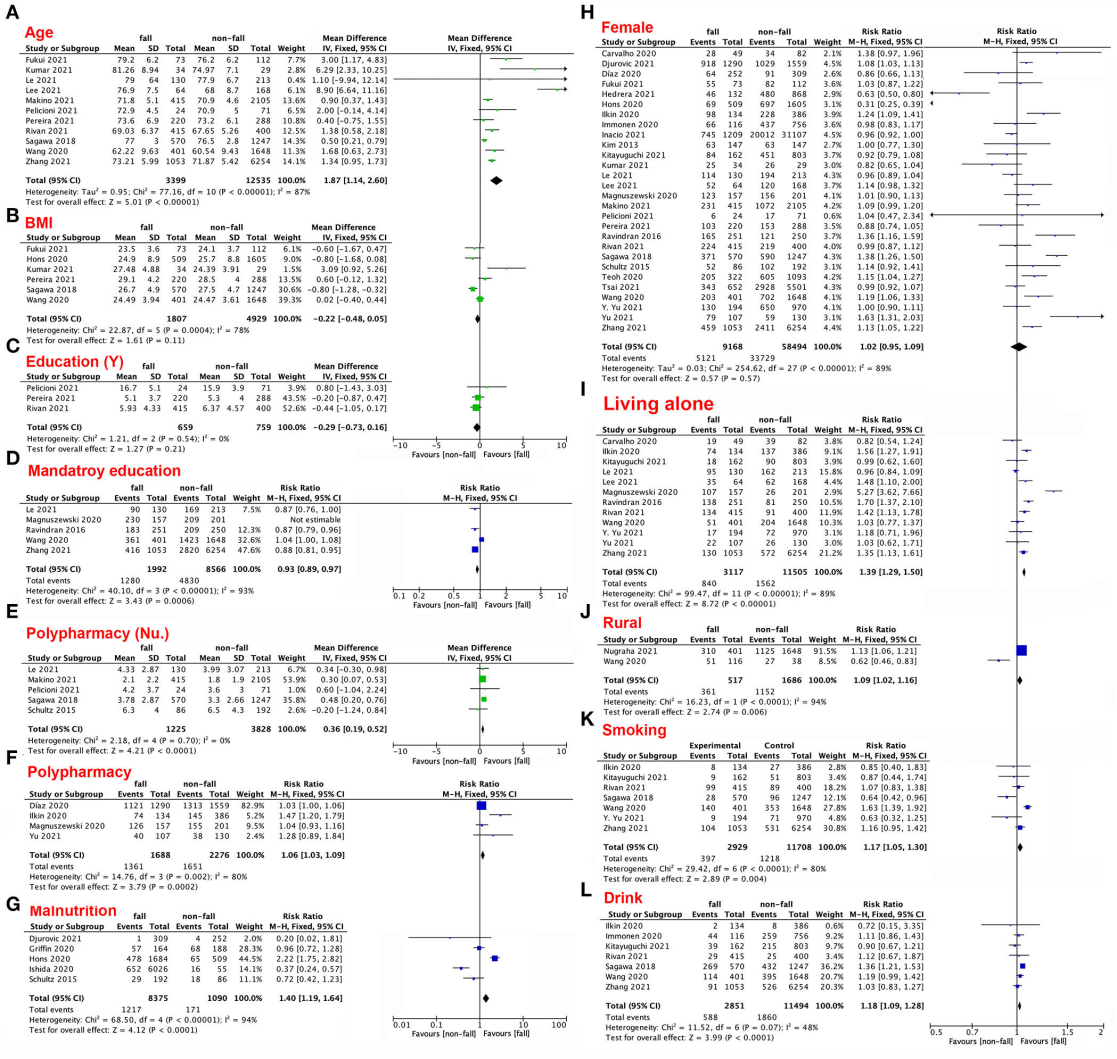
**(A–L)** Forest plots of the impacts of patient characteristics and lifestyle factors on the risk of falls.

First, we compared aging adults in terms of age, BMI, education level, polypharmacy, malnutrition, sex (female), living alone, living in a rural area, smoking status, and alcohol consumption (Figures 2A–L). Older age (MD 1.87; 95% CI 1.14–2.6; *p* < 0.00001, Figure 2A), number of drugs used (MD.36; 95% CI.19–0.52; *p* < 0.0001, Figure 2E), and polypharmacy (RR 1.06; 95% CI 1.03–1.09; *p* = 0.0002, Figure 2F) were associated with increased incidence of falls. Malnutrition (RR 1.4; 95% CI 1.19–1.64; *p* < 0.0001, Figure 2G), living alone (RR 1.39; 95% CI 1.29–1.5; *p* < 0.00001, Figure 2I), living in a rural area (RR 1.09; 95% CI 1.02–1.16; *p* = 0.006, Figure 2J), smoking (RR 1.17; 95% CI 1.05–1.3; *p* = 0.004, Figure 2K), and alcohol consumption (RR 1.18; 95% CI 1.09–1.28; *p* < 0.001, Figure 2L) were risk factors for falls. Education level (MD −0.29; 95% CI −0.73–0.16; *p* = 0.21, Figure 2C) had no impact on risk of falls, but completion of the mandatory level of education (RR 0.93; 95% CI 0.89–0.97; *p* = 0.006, Figure 2D) decreased the risk of falls. BMI (MD −0.22; 95% CI −0.48–0.05; *p* = 0.11, Figure 2B) and sex (RR 1.02; 95% CI 1–1.04; *p* = 0.13, Figure 2H) did not affect risk of falls.

### Comorbidities in people with or without falls

**Figure 3:**
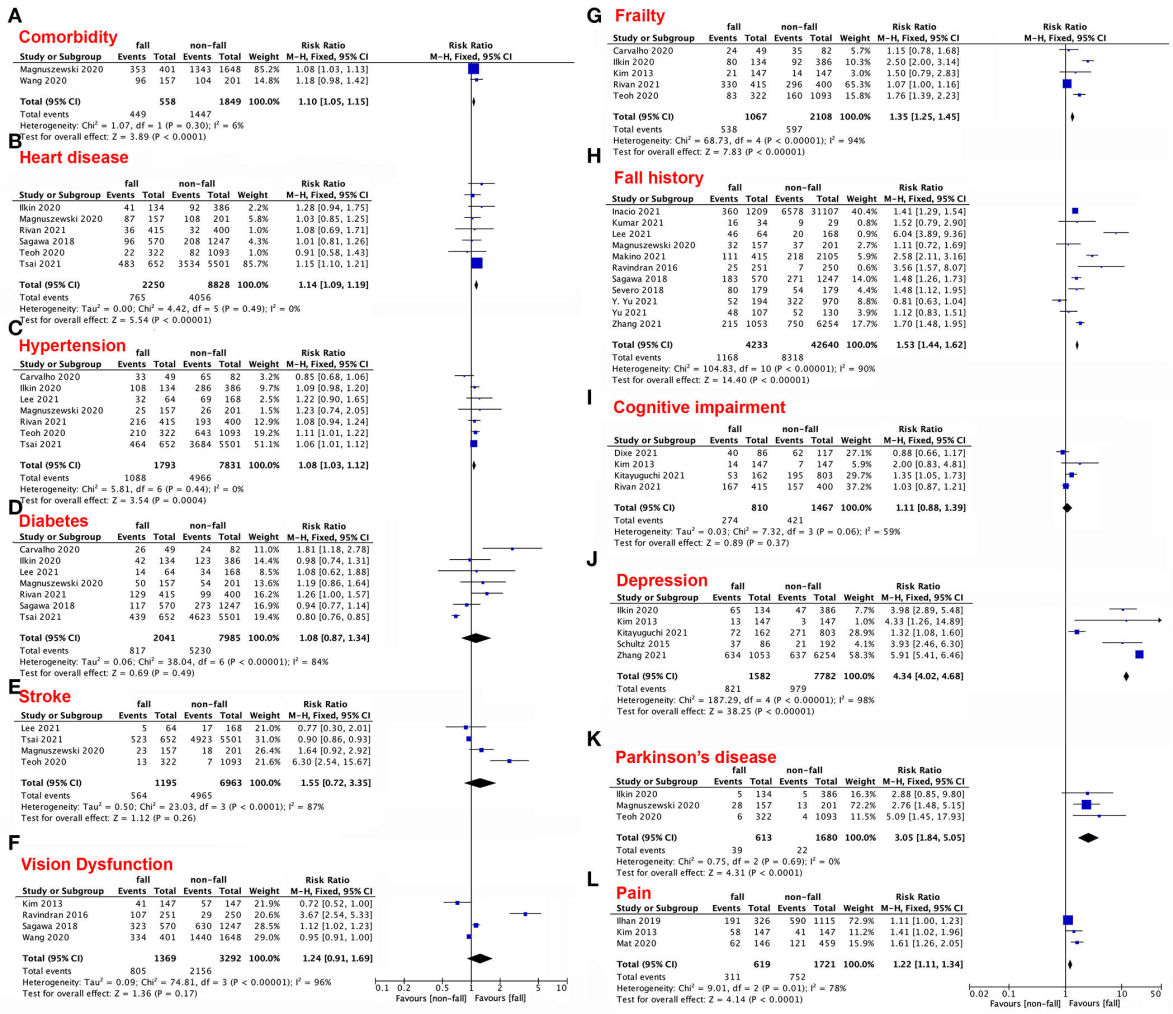
**(A–L)** Forest plots of the impacts of comorbidities on the risk of falls.

Eleven comorbidities were compared between people with and without falls: cardiac disease, hypertension, diabetes, stroke, vision dysfunction, frailty, fall history, cognitive impairment, depression, Parkinson's disease, and pain (Figures 3A–L). Even though these comorbidities may alter the rate of frailty among elderly individuals (RR 1.1; 95% CI 1.05–1.15; *p* < 0.0001, Figure 3A), not all of the comorbidities mentioned above necessarily influence falls. For instance, diabetes (RR 1.08; 95% CI 0.87–1.34; *p* = 0.49, Figure 3D), stroke (RR 1.55; 95% CI 0.72–3.35; *p* = 0.26, Figure 3E), vision dysfunction (RR 1.24; 95% CI 0.91–1.69; *p* = 0.17, Figure 3F), and cognitive impairment (RR 1.11; 95% CI 0.88–1.39; *p* =0.37, Figure 3I) did not significantly differ between the two groups. In contrast, heart disease (RR 1.14; 95% CI 1.09–1.19; *p* < 0.00001, Figure 3B), hypertension (RR 1.08; 95% CI 1.03–1.12; *p* = 0.0004, Figure 3C, frailty (RR 1.35; 95% CI 1.25–1.45; *p* < 0.00001, Figure 3G), fall history (RR 1.53; 95% CI 1.44–1.62; *p* < 0.00001, Figure 3H), depression (RR 4.34; 95% CI 4.02–4.68; *p* < 0.00001, Figure 3K), Parkinson's disease (RR 3.05; 95% CI 1.84–5.05; *p* < 0.0001, Figure 3K), and pain (RR 1.22; 95% CI 1.11–1.34; *p* < 0.0001, Figure 3L) were associated with increased risk of falls among the aging population.

## Discussion

In older adults, falls impose major health, economic, and societal burdens (16). Falls are the leading cause of injury in the elderly population (36). A serious fall could result in decreased independence and reduced QOL (36). Hip fracture, in particular, is a serious and devastating consequence of falling in older individuals (36). Moreover, Makino et al. reported that fall history is the most influential predictor of future falls (25). According to recent research, fall history increases the current risk of falls. Some research has also proposed that fear of falling is significantly associated with falls. Usually, fear of falling arises from a fall history (45). Patil R et al. suggested that fear of falling may increase even after a non-injurious fall. Subsequently, older adults may enter into a negative cycle in which they reduce their activity, leading to reduction in functionality (45). To avoid this negative cycle, we recommend early prevention of falls in elderly adults. Fear of falling was also independently associated with presence of knee pain, with a significant relationship observed between fear of falling and moderate to severe knee pain but not mild knee pain (14). Pain is a frequently mentioned factor, but only a few studies have prospectively collected data on fall occurrence in relation to knee pain or the lack of association between knee pain and fall occurrence during long-term follow-up. Furthermore, fear of falling may exacerbate depression. Our present results demonstrated that depression can also impact the risk of falls. As most falls result from loss of balance while walking and poor balance is the leading risk factor for falls, people tend to focus on the importance of mobility in the risk of falls (46). This explains the lack of sufficient predictive factors in older adults at risk of one or more falls. Additionally, social factors can increase the psychological burden on elderly individuals and reduce self-care capability, a factor with strong influences (47) on the risk of falls as well as the incidence rates of many diseases. Thus, the identification of risk factors for falls will provide important guidance for the care of elderly individuals.

Older age, polypharmacy, malnutrition, frailty, smoking, and alcohol consumption significantly increased the risk of falls; these factors also reflect decline in physical condition. Moreover, chronic illnesses are very common in older adults, and cardiac disease, hypertension, diabetes, stroke, and Parkinson's disease are associated with falls. Older adults residing in urban areas had a higher risk of falling than those residing in rural areas (27). This difference may be explained by traffic, which can impede medical treatment. Residency in suburban areas has certain advantages; for instance, it is easier to engage in physical exercises, such as walking, in suburban and rural areas than in urban areas. Physical exercise helps to reduce the risk of falls in adults and improves lower limb strength in older people (27, 47). Moreover, living in a rural area is associated with less pollution exposure; this factor is particularly important in developing countries because pollution may cause comorbidities. However, only a few articles have focused on this topic. We plan to explore this topic further in the future once a larger number of relevant reports have been published. Sex has been identified as a risk factor for falls among older adults (37), but in our study, women did not have a higher risk of falling than men. While women experience a higher rate of frailty than men (37), men are more likely to exhibit harmful lifestyle habits, such as smoking and consuming alcohol; therefore, sex differences in the risk of falling merit further study. Another risk factor in our study is living alone, which increases the risk of depressive symptoms and the impacts of falls.

A major strength of this study is that we analyzed data from several large-scale, well-characterized cohorts and systematically summarized the risk factors for falls in the elderly population. These findings can inform healthcare in the elderly population. Biswas et al. explored the risk factors for falls among older adults in India (6); however, their study focused on only the Indian population and thus exhibited geographic and ethnic limitations. Xie et al. examined risk factors for the development of fear of falling, but fear of falling was only one of the risk factors for falls; we suggest that it is more meaningful to identify the risk factors for falls. Our meta-analysis also has some limitations. For example, we did not categorize the participants according to whether they lived in the community or in nursing homes, which is a major factor associated with the risk of falls.

## Conclusion

We demonstrated that (1) older age, polypharmacy, malnutrition, single status, living in a rural area, smoking, and alcohol consumption significantly increased the risk of falls in elderly adults. In contrast, higher education level was protective against falls. Additionally, we found that (2) individuals with cardiac disease, hypertension, frailty, previous history of falls, depression, Parkinson's disease, and pain had a higher risk of falls than individuals without such comorbidities.

## Data availability statement

The original contributions presented in the study are included in the article/Supplementary material, further inquiries can be directed to the corresponding authors.

## Author contributions

Data acquisition and drafting of the manuscript: QX, XO, and JL. Conception and design of the study: JL. Analysis and/or interpretation of data: QX and XO. All authors contributed to the article and approved the submitted version.

## Conflict of interest

The authors declare that the research was conducted in the absence of any commercial or financial relationships that could be construed as a potential conflict of interest.

## Publisher's note

All claims expressed in this article are solely those of the authors and do not necessarily represent those of their affiliated organizations, or those of the publisher, the editors and the reviewers. Any product that may be evaluated in this article, or claim that may be made by its manufacturer, is not guaranteed or endorsed by the publisher.
