# Supplementary material for: The risk of falls among the aging population: A systematic review and meta-analysis
Source: Front Public Health. 2022 Oct 17;10:902599. doi: 10.3389/fpubh.2022.902599 (PMC9618649; doi:10.3389/fpubh.2022.902599)
Supplement: Supplementary file 1 [file Table_1.DOC]

| **Subject headings** | **Keyword search** |
| --- | --- |
| Risk factors/Predicting Factors/Predictor | “Risk factors” OR “Predicting factors” OR “Predictor demographic characteristics” OR “Lifestyle factors” OR “Comorbidities” |
| Demographic characteristics | “Age” OR “Old” OR “Body Mass Index” OR “BMI” OR “Weight” OR “Fat” OR ”Education” OR “Polypharmacy ” OR “Multiple Medicine” OR “Multiple Drug” OR “Malnutrition” OR “Undernutrition” OR “Malnourished” OR “Undernourished” OR “Gender” OR “Female” OR “Sexual” |
| Lifestyle factors | “Living along” OR “Single” OR “Lonely” OR “Marriage” OR “Living in Rural” OR “Living in suburb” OR “Smoking” OR “Drink” OR “Intemperance” OR “Alcohol Addiction” OR “Alcohol consumption” |
| Comorbidities | “Comorbidity” OR “Disease” OR “Accompanying disease” OR “Concomitant disease” OR “Heart disease” OR “Heart trouble” OR “Cardiovascular Disease” OR ”Cardiopathy” OR “Hypertension” OR “High blood pressure” OR “Diabetes” OR “Diabetes mellitus” OR “Stroke” OR “Palsy” OR “Vision Dysfunction” OR “Visual dysfunction” OR “Frailty” OR “Weak” OR “” OR “Asthenia” OR “Fall History” OR “Cognitive impairment” OR “Cognitive dissociation” OR “Depression” OR “Stress” OR “Nervous” OR “Parkinson disease” OR “Neuropathy” OR “Mental disorder” OR “Neurological disorders” OR “Pain” OR “Miserable” |
| Fall/Drop | “Fracture” OR “Trip OR Drop” OR “Fall” OR “Imbalance” |

**Supplementary File 1: Complete detail of search strategy**

Searching title and abstract
